# Emergent microenvironments of nucleoli

**DOI:** 10.1080/19491034.2024.2319957

**Published:** 2024-03-05

**Authors:** Matthew R. King, Kiersten M. Ruff, Rohit V. Pappu

**Affiliations:** Department of Biomedical Engineering and Center for Biomolecular Condensates, Washington University in St. Louis, Campus, MO, USA

**Keywords:** Biomolecular condensates, emergent properties, nucleolus, pH gradients, phase separation

## Abstract

In higher eukaryotes, the nucleolus harbors at least three sub-phases that facilitate multiple functionalities including ribosome biogenesis. The three prominent coexisting sub-phases are the fibrillar center (FC), the dense fibrillar component (DFC), and the granular component (GC). Here, we review recent efforts in profiling sub-phase compositions that shed light on the types of physicochemical properties that emerge from compositional biases and territorial organization of specific types of macromolecules. We highlight roles played by molecular grammars which refers to protein sequence features including the substrate binding domains, the sequence features of intrinsically disordered regions, and the multivalence of these distinct types of domains / regions. We introduce the concept of a barcode of emergent physicochemical properties of nucleoli. Although our knowledge of the full barcode remains incomplete, we hope that the concept prompts investigations into undiscovered emergent properties and engenders an appreciation for how and why unique microenvironments control biochemical reactions

## Introduction

Nucleoli are micron-sized membraneless organelles that have captured the attention of microscopists since the 1700s [1]. Foundational electron microscopy revealed that nucleoli in higher metazoans comprise three nested regions, each of different densities [2]. These regions harbor distinct macromolecular compositions and represent spatial inhomogeneities that span length scales that are one to two orders of magnitude larger than the dimensions of the underlying molecules. These features suggest that nucleoli represent distinct phases within the nucleoplasm and nucleolar regions of specific densities and compositions are coexisting sub-phases [3].

Distinct phases, such as nucleoli coexisting with the nucleoplasm or coexisting sub-phases within nucleoli can come about through that a combination of processes. These include: (i) phase separation, which is a segregative density and / or compositional transition [4,5]; (ii) reversible associations that include site-specific binding, which gives rise to complexes of precise stoichiometries [4,5]; (iii) polyelectrolyte complexation, which involves associations among oppositely charged macroions to form a range of complexes that can undergo phase separation [6–11]; and (iv) percolation, also known as gelation, which is a continuous phase transition that gives rise to macromolecular networks defined by a range of stoichiometries that form due to a multivalence of specific interactions [4,12]. We refer to the totality of processes that give rise to distinct coexisting phases as *macromolecular condensation* [4]. Note that these are concentration-dependent processes and at ultra-low concentrations, phase separation may not be operative, but the other processes that contribute to condensation will be operative [13–16]. Here, we shall focus on emergent properties that result from the totality of processes that come under the rubric of condensation.

Depending on the types of macromolecules that are differently, and nonrandomly organized within a condensate, the intrinsic physicochemical properties of the macromolecules can become generators of emergent interphase and interfacial properties [17]. For example, if the macromolecules that are concentrated in a dense phase have a net charge, then we would expect an uneven distribution of cations and anions across the phase boundary that maintains electroneutrality in both the dilute and dense phases and gives rise to an interphase Donnan potential [18]. This and other interphase potentials are emergent properties that derive from the formation of distinct coexisting phases. Other interphase properties, which might even generate interphase electrochemical potentials, include passive gradients of pH, susceptibilities including dielectric responses, and hydrophobicity. These will have temporal variations that will be influenced by differences in transport/material properties such as viscosity, and viscoelasticity [8,19–26]. At the interface between condensates and the coexisting cellular milieu or at interfaces between internal coexisting sub-phases, as would be the case in nucleoli, there will be an accumulation of functional groups that prefer to localize to interfaces. These groups will contribute to distinct properties such as interfacial tension, interfacial redox potentials or electric potentials, and differential accumulation of apparently hydrophobic versus hydrophilic groups at the interface [27–39]. The key takeaway is that the totality of processes that come under the rubric of macromolecular condensation can generate distinct internal microenvironments and interfaces defined by distinct physico-chemical properties when compared to the surrounding nucleoplasm. These emergent internal interphase properties as well as interfacial characteristics will be governed by the densities, compositions, and the nonrandom territorial organization of macromolecules that are specific to the body of interest, which in our case is the nucleolus.

**Figure 1.**
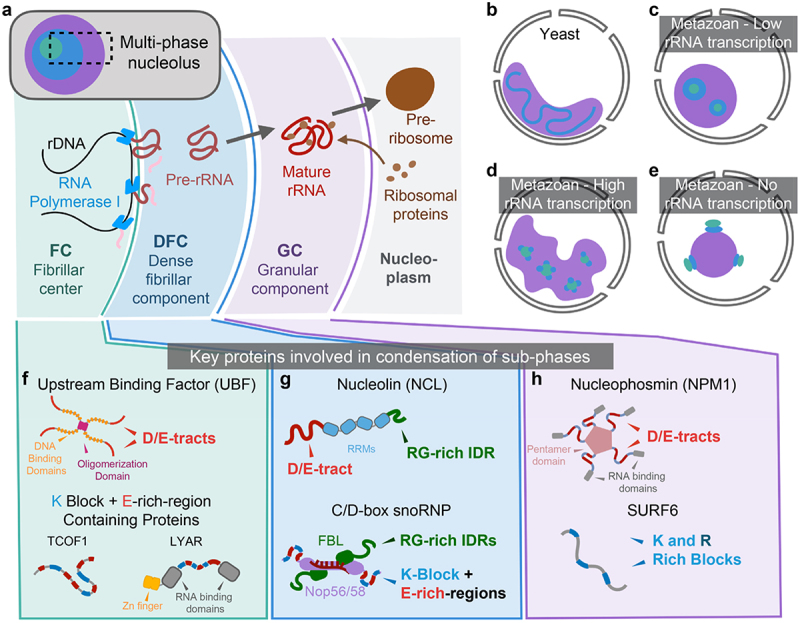
Nucleoli are hierarchically organized into sub-phases. (a) schematic of ribosomal assembly in the nucleolus in higher metazoan eukaryotes. The three distinguishable phases observed by electron microscopy are the fibrillar center (FC), the dense fibrillar component (DFC) and the granular component (GC). The outward flux of transcribed ribosomal RNA (rRNA) is met by a countervailing inward flux of ribosomal proteins (rProteins) in the GC. (b) yeast nucleoli exhibit two phases. (c) Nucleoli in low rRNA transcribing cells tend to have a spherical shape and smaller subphases relative to more active cells. (d) Nucleoli in high rRNA transcribing cells tend to have larger FCs, a greater number of DFCs, and larger and more amorphous GCs than quiescent cells. (e) Nucleoli that have been drugged with transcriptional inhibitors exhibit ‘inside out’ morphology with FC sub-phases exposed to the nucleoplasm and capping DFC sub-phases, which in turn, are nestled between these FC caps and a large single GC sub-phase. (f-h) the key proteins known to contribute to condensation of sub-phases. (f) key FC proteins are upstream binding factor (UBF) and K block + E-rich-region IDR containing proteins such as Treacher Collins associated factor 1 (TCOF1) and ly-1 antibody reactive (LYAR). (g) key DFC proteins are nucleolin (NCL) and fibrillarin (FBL) contained in the C/D box snoRNP. (h) key GC proteins are nucleophosmin (NPM1) and Lysine/Arginine (K/R)-block contain IDRs such as SURF6.

The most well-known functions of nucleoli are processes involved in ribosome biogenesis, and these are thought to occur in an assembly-line-like manner [40,41]. Numerous lines of evidence suggest that nascent ribosomal RNA molecules make up an outward flux from their site of transcription at the interface between the fibrillar center (FC) and the dense fibrillar component (DFC). Correspondingly, ribosomal proteins are thought to form an inward flux from the nucleoplasm into the granular component (GC), where pre-ribosome assembly takes place Figure 1(a) [3,40].

The setting up of complementary fluxes is likely an emergent consequence of nonrandom macromolecular organization that is distinct from the nucleoplasm and distinct across nucleolar sub-phases [42,43]. The underlying compositional organization of nucleolar sub-phases is coming into focus, thanks largely to high-throughput microscopy [44,45]. A recent study leveraged these datasets to show that the distinct compositional biases of intrinsically disordered regions (IDRs) of proteins that localize to the FC, DFC or GC creates a proton gradient radiating inward from the nucleoplasm into the nucleolus, with the FC/DFC being more acidic than the outer rim of the GC [46]. The presence of a proton gradient could set up a proton motive force [47] to help facilitate the creation of opposing fluxes in active nucleoli. This proton motive force is approximately -88 mJ/proton based on the measured pH gradient.

Motivated by recent findings, we review the determinants of nucleolar organization, including the scaffold macromolecules and functions of the sub-phases. We also review and expand upon recent discoveries showing how compositional biases distinguish nucleoli from other nuclear bodies, as well as nucleolar sub-phases from each other. Of particular interest are the molecular grammars of nucleolar IDRs. We offer conjectures regarding emergent physicochemical properties that are to be expected based on the compositional and intra-nucleolar territorial biases that we are beginning to learn about. In doing so we strive to lay the groundwork for understanding how emergent microenvironments of nucleoli arise and how these might contribute to the multitude of nucleolar functions.

## Nucleolar organization

It is now thought that specific nucleolar factors are drivers of condensation that give rise to nucleolar sub-phases. This in turn enables key factors to be selectively partitioned to the sub-phase where they function [3]. Ribosomal DNA and the transcriptional machinery are housed in the FC. Premature ribosomal RNA is transcribed at the interface between the FC and the DFC. Ribosomal RNA (rRNA) is, for the most part, folded, spliced, chemically modified, and complexed with ribosomal proteins as it progresses through the DFC and the GC into the nucleoplasm Figure 1(a).

A combination of investigations based on genetics, biochemistry, and high-throughput cryo-EM have yielded high-resolution models of the structured aspects of the complex process of ribosome biogenesis [48–50]. By and large, these models are built on ribosome assembly intermediates isolated from *Saccharomyces cerevisiae* [51]. This is relevant because many aspects of ribosome biogenesis that are accessible to investigations via the tools of structural biology appear to be conserved in higher eukaryotes [52]. The most up-to-date *in situ* evidence supports assembly-line like models in yeasts (Figure 1b) and higher eukaryotes (Figure 1c,d), where ribosome biogenesis is thought to occur as transcripts form a radially outward flux from their site of transcription at the FC/DFC interface [53–57]. Here, we focus on nucleoli from higher metazoans that have nested FC, DFC, and GC sub-phases [58,59].

**Figure 2.**
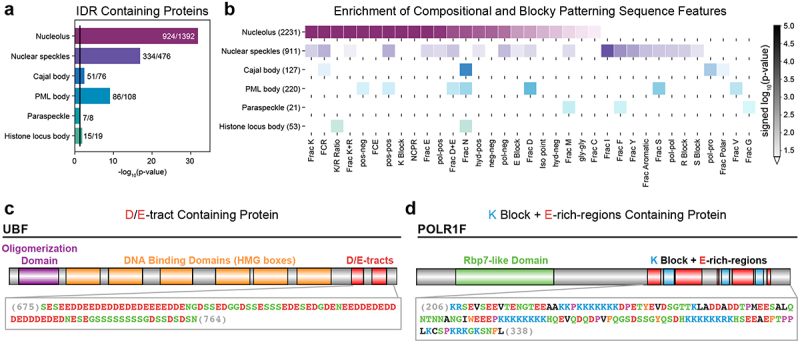
Nucleoli are enriched in IDRs that have specificmolecular grammars. (a) p-values quantifying the enrichment of IDR containing proteins within major nuclear bodies (Fisher’s exact test). The black line denotes a p-value cutoff of 0.05. The human protein atlas was used to extract proteins localized to each of the nuclear bodies. The first number reports the number of IDR containing proteins and the second number reports the number of proteins analyzed per nuclear body. (b) Summary of compositional and blocky patterning sequence features that are enriched in different nuclear bodies. Patterning parameters are denoted by ‘x-y’, where x and y are the two amino acid types whose patterning with respect to each other are being quantified. Numbers in parentheses denote the number of IDRs analyzed within each nuclear body. p-values were calculated using the two-sample Kolmogorov-Smirnov test comparing z-score distributions from a particular nuclear body to the remaining IDRome. (c) Domain architecture of an example D/E-tract containing protein, UBF. (d) Domain architecture of an example K block + E-rich-region containing protein, POLR1F. In (c) and (d) the relevant IDR sequences are shown and color-coded by amino acid type.

Detailed maps of the steps of ribosome biogenesis that are accessible to structural techniques are contributing to an understanding of how specific molecular-scale nucleolar processes occur [50]. However, structural probes cannot provide insights regarding emergent microenvironments of sub-phases. These are governed by the totality of macromolecular compositions and require direct measurements of physicochemical properties and the consideration of regions that are inaccessible to structural annotations, specifically IDRs of nucleolar proteins. Indeed, the nucleolus harbors a disproportionally high fraction of IDRs (Figure 2a), many of which are essential for nucleolar functions [60–62].

The discoveries that signal recognition particles are synthesized in mammalian nucleoli [63,64] and that the majority of resident nucleolar proteins are not involved in ribosome biogenesis [65,66] were critical in establishing the new view of the nucleoli being plurifunctional [2]. The growing list of nucleolar functions includes DNA repair, recombination, transcription, and telomere maintenance. It is thought that depending on the cell type and cell state, the nucleolus can be a central hub that facilitates many complex biochemical processes simultaneously or serve as a repository for factors that are mostly latent or need to be sequestered [67,68].

The functional diversity of nucleoli does not fully impair the efficiency of ribosome biogenesis. So, how is a combination of functional diversity and efficiency of each of the functions achieved? Given the invocation of condensation as a driver of nucleolar assembly and regulated dissolution, the question that arises is if the microenvironments of the FC, DFC, GC, and other sub-phases are unique and if they affect biochemical processes? The GC of the nucleolus has liquid-like properties. This means that the GC is dominantly viscous rather than elastic over the timescales of interest and functional relevance. Perturbations that lead to slower internal reorganization of,key nucleolar proteins, which may be due to increased elasticity, are correlated with reduced rRNA production [27,34,69,70]. Furthermore, *in vitro* reconstitutions suggest that the capacity for hierarchical organization is encoded in key factors that drive condensation. Minimal condensates organized around GC components *in vitro* show how fluxes and selective partitioning of ribosomal proteins can be achieved [42,43,71–73]. These results underscore the importance of understanding nucleolar functions in the context of condensation. In reviewing the compositional biases of nucleolar IDRs, our goal is to highlight the physicochemical properties of nucleolar microenvironments that can emerge because of these biases.

## Nucleolar sub-phases are defined by distinct macromolecular compositions

The inventory of DNA loci, RNAs, and proteins that reside in the nucleolus has recently been significantly expanded by unbiased, high-throughput studies using microscopy, proteomics, and genomics tools [44,45,74–77]. At the core of nucleoli are nucleolar organizing centers (NORs), which are the centromere-proximal tandem arrays of rDNA genes found on five chromosomes. A NOR and its resultant rRNA transcripts dominate the nucleotide composition of an active nucleolus [3]. Still, the nucleolus interacts dynamically with many non-NOR chromatin loci [78] and houses a rich composition of RNAs wherein even low-abundance long non-coding RNAs (lncRNAs) play roles in maintaining nucleolar morphology [73] and accurate rRNA transcription [79].

Protein compositions of nucleoli have been evaluated via mass spectroscopy of isolated nucleoli [66] and more recently by high-throughput microscopy, often coupled with proteomics [75]. These latter efforts, termed spatial proteomics, have tallied up to 1425 nucleolar proteins (https://www.proteinatlas.org/humanproteome/subcellular/nucleoli). While there are inherent limitations to these global studies – for example the FC and DFC cannot be distinguished in these datasets due to limits on spatial resolution – they nonetheless provide a roadmap for more targeted surveys. For example, using high-resolution microscopy, a recent study mapped over 200 nucleolar proteins suspected to be involved in ribosome biogenesis, each tagged with green fluorescence proteins (GFPs). This study led to the discovery of a unique region of the nucleolus termed the peripheral DFC or PDFC. The PDFC appears to be involved in processing 3' untranslated regions (UTRs) of rRNAs [56]. Complementary efforts, based on high-throughput microscopy, are allowing for the screening of factors that coordinate the numbers and sizes of nucleolar sub-phases [80]. Overall, the rich and continually growing atlas of proteins localized to the nucleolar sub-phases represents an invaluable resource for investigating nucleolar composition. These inventories create an opportunity to organize information in a way that allows for improved understanding of how distinct compositional biases in FCs, DFCs, GCs, and other sub-phases become generators of distinct microenvironments and functions. To make this point, we provide a survey of key factors that have been shown to affect sub-phase condensation and ribosome biogenesis.

Ribosomal DNA and the transcription factor UBF (upstream-binding factor) together serve as the nidus of the FC (Figure 1a). Loss of either component results in mis-condensation of nucleolar factors away from rDNA *in vivo* [81,82]. Further, *in vitro* reconstitutions of FC-like condensates require only rDNA, UBF, and at least one FC-localized protein containing IDRs that feature blocks of lysine residues (K-blocks) that are interspersed by regions rich in glutamic acid residues [46]. Therefore, rDNA, UBF, and proteins containing IDRs featuring K-blocks and E-rich-regions appear necessary and sufficient to drive FC formation. Interestingly, it was recently shown that transgenic over-expression of the 150-kDa Treacle/Treacher Colins Factor 1 (TCOF1) in normally FC-free zebrafish was sufficient to generate an FC-like compartment in those animals, as assessed by osmium tetroxide/uranyl acetate staining and electron microscopy [83]. TCOF1 is predicted to be entirely disordered and consists almost exclusively of K blocks and E-rich-regions. Increasing the copy numbers of UBF in *Xenopus* oocytes via mRNA injection leads to enlarged oocytes, which corroborates its established role as an FC scaffold. Increasing the copy numbers of TCOF1 can result in a similar phenotype, but only if mRNA loads are at least 5-times higher than that of UBF [46]. These findings suggest that TCOF1 expression, which is spatiotemporally regulated to facilitate ribosome biogenesis in fast growing cells such as the developing neural crest, could be used to tune FC size and possibly rRNA output. Among the FC proteins that feature K-blocks and E-rich-regions are RNA polymerase I and Topoisomerase I, as well as the FC and DFC enriched proteins nuclear casein kinase and cyclin-dependent kinase substrate (NUCKS) and ly-1 antibody reactive (LYAR) [46,84] (Figure 1f).

Investigations of the rDNA chromatin state, enabled by the recent complete annotation of the human genome [85], point to UBF (upstream-binding factor) as being bound throughout the cell cycle and as being a key to nucleolar reformation following mitosis [86]. Additionally, studies of rDNA chromatin are revealing its role in nucleolar morphology and function across cell types and cell states [54,87–89]. For example, evidence from different hematopoietic cells that range in ribosomal output by over an order of magnitude suggests that larger FC sizes and a specific chromatin landscape at NORs are correlated with increased ribosomal outputs [58,59,90]. The FC sits at the core of ribosome biogenesis, and there appear to be clear compositional biases that delineate the FC from other nucleolar sub-phases. The totality of emergent physicochemical properties that derive from the distinct compositional biases of FCs and how they impact rRNA transcription at the FC/DFC interface are topics of significant interest [49].

The morphology of the DFC *in vivo* is strongly coupled to rRNA transcription. As a distinct sub-phase, the main constituents of DFCs are pre-mature rRNA (pre-rRNA) and RNA-binding proteins involved in rRNA folding and maturation [62,76]. DFCs are more numerous in cells that produce higher amounts of rRNA. During transcriptional inhibition DFCs collapse into a few large ‘caps’ that appear on the surface of a GC condensate [3] (Figure 1c-e). Interfaces between condensates are governed by differential solvation preferences of the macromolecular components [27,28,91]. A core-shell architecture of a condensate implies that the components of the core are relatively more hydrophobic than the components of the shell or that the components of the shell are more hydrophilic than those of the core. The rRNA-dependent alterations of core-shell organization highlights how rRNA levels contribute to the differential solvation preferences, which refers to the relative hydrophobicity/hydrophilicity of protein-RNA mixtures. Further, at least three categories of non-rRNA factors have been shown to play a role in DFC condensation. These factors are Arg (R) and Gly (G) rich (RG-rich) IDRs present in C/D box and H/ACA snoRNPs [27,92], the rRNA chaperones nucleolin (NCL) and DDX21 [46,73], and the lncRNAs SLERT and LoNA [57,93,94] (Figure 1g). All these factors are involved in key functions of the DFC, which include the proper folding, splicing, and chemical modification of rRNA.

Extant evidence suggests that pre-rRNA and abundant chaperones with multivalent RNA binding sites, such as NCL, contribute to the driving forces for DFC condensation [27,46,73]. Furthermore, DFC maintenance and integrity, as assessed by the mis-localization of canonical DFC components like fibrillarin, appears to be controlled by the RG-rich IDRs and to a lesser extent by lncRNAs [57,95]. Work with *in vitro* reconstituted DFC facsimiles suggests that the relative hydrophobicity of this condensate leads to it being wetted onto and encased by the FC and GC condensates, respectively [27,46,73]. The DFC is a distinctively responsive condensate that features diverse compositional biases while serving as a hub for diverse biochemical reactions that can remodel its morphology and enable responsiveness to ribosome production (Figure 1c-e). Thus, as a condensate, the DFC provides rich opportunities for studying general principles about relationships among biochemical activity, morphology, and contributions to differential solvation.

The GC is the largest nucleolar sub-phase, and it is thought to be scaffolded by the abundant chaperone nucleophosmin (NPM1) and proteins with IDRs that feature blocks of Lys and Arg residues, such as Arf1, SURF6, and ribosomal proteins [27,72] (Figure 1h). A model that links features of GC phase separation to its function, facilitating ribosome biogenesis, is the so-called ‘molecular hand-off model’ [43,72]. In this model, the inward flux of ribosomal proteins from the nucleoplasm into the GC is complemented by the outward flux of rRNA from the DFC into and through the GC. Work to date has focused on how the inward flux of ribosomal proteins (rProteins) from the nucleoplasm into the GC is mediated by NPM1 [43]. Furthermore, recent findings suggest that the phase equilibria of rRNA-NPM1 condensates are synergistically linked to the binding equilibria of rRNAs and rProteins, such that radially inward fluxes of rProteins into the rRNA-NPM1 condensates are complemented by radially outward fluxes of rRNA into the GC that drive ribosomal assembly and trafficking of ribosomes into the nucleoplasm [33]. However, many details of this handoff model remain unclear, and one might need to go beyond simple thermodynamic considerations to arrive at deeper explanations for how fluxes are set up in opposing directions [96]. A more complete model of GC will need to incorporate the mechanisms of how rRNA fluxes from the DFC into and through the GC are established. Due considerations are also needed of other GC-localized ribosome assembly chaperones and the observed RNA-rich and protein-rich territoriality of the GC [64,97,98].

As is true for all biomolecular condensates, a comprehensive model of FC, DFC or GC condensation and integrity will require systematic and unbiased studies, such as the ones that have been carried out for cytoplasmic stress granules that arise from sodium arsenate poisoning [99–101]. In this context, it is worth noting that such studies will require creative strategies since complete knockout of condensate scaffolds (e.g., UBF and NCL) are lethal [82,102]. Nevertheless, surveying the established drivers of nucleolar condensation indicates an important role played by proteins that contain IDRs. Using the classifications of proteins from the Human Protein Atlas, it was shown that IDR containing proteins are enriched in nuclear bodies compared to the remaining human proteome (Figure 2a) [62]. Nucleolar proteins show the highest degree of enrichment in IDR containing proteins compared to the rest of the nuclear bodies (Figure 2a). This over-representation suggests that IDRs may play an especially important role in nucleolar function/organization, and this is suggestive of the possibility that the molecular grammars of IDRs can encode essential interactions that give rise to emergent properties of nucleoli.

## Molecular grammars of nucleolar IDRs

Molecular grammars of IDRs are defined by nonrandom compositional biases and linear sequence patterns [46,103–106]. Previous studies have shown that the driving forces for IDR-mediated condensation derive from distinct molecular grammars. Known grammars include the valence and patterning of aromatic residues [107–109], covariations of Tyr and Phe as well as Ser and Gly [108], distinct sequence blocks rich in aromatic and Arg residues [110], negatively charged IDRs that complex with positively charged macroions [35], blocks of oppositely charged residues [111], mixed charge domains featuring regular repeats of Arg and Asp residues or Arg and phosphorylated Ser residues [112], IDRs rich in RG motifs [113–115], protein–protein interaction networks organized around prion-like low complexity domains with glutamine-rich regions [116], and the presence of clusters of aliphatic residues [117,118]. These molecular/sequence grammars contribute to driving forces for condensation and do so either via homotypic or heterotypic interactions or a combination of the two types of interactions [101,119]. Besides contributing to the driving forces for condensation, molecular grammars of IDRs have also been shown to contribute to the material properties of condensates, as well as networks of functional heterotypic interactions that determine selective partitioning and recruitment of substrates into condensates [110,120].

The concept of molecular grammars, first introduced by Wang et al., in 2018, which built on the works of others [27,28,35,115,121–125], highlights the importance of unique physicochemical contributions of amino acid residues, as well as short linear motifs [126] and the multivalence of these motifs. Studies that have highlighted the role of molecular/sequence grammars in condensation and functional interactions of condensates demonstrate the importance of small differences whereby seemingly similar chemistries such as Ser versus Thr, Asn versus Gln, Glu versus Asp, Tyr versus Phe versus Trp, or Arg versus Lys can contribute very differently to condensate formation, the material properties of condensates, and the functional interactions of condensates [107,108,110,112,121]. Similar considerations apply when studying purine vs. pyrimidine rich regions in RNA sequences [39,127,128]. Importantly, compositional biases and sequence patterns are often evolutionarily conserved [105]. Accordingly, the presence of functionally relevant interactions is decipherable using suitable evolutionarily analyses. However, traditional sequence alignment methods are inapplicable for analyzing IDRs and unmasking evolutionarily important and functionally relevant molecular grammars. This has catalyzed the development of new methods that leverage work done over the past decade-and-a-half that has helped uncover how compositional biases and sequence patterns influence the conformational preferences and functional interactions of IDRs. These investigations, inspired by findings regarding sequence-ensemble relationships of IDRs [129–138], have given rise to codifiable insights, which now undergird algorithms that are used for evolutionary analyses of IDRs [105,106,133]. Here, we focus our description of an algorithm developed in our lab, which we refer to as NARDINI+ [46,103,139].

NARDINI+ is an algorithm that unmasks nonrandom compositional biases and linear sequence patterns within IDRs. Sequences are represented as feature vectors. Each component in the feature vector is a z-score that quantifies either the extent of enrichment of a compositional feature compared the rest of the IDRome of interest, or the degree of linear segregation versus mixing of pairs of residue types when compared to random sequences that have identical compositions.

Distinct nuclear bodies are defined by compositional specificities of their IDRs, and this is readily demonstrated in the analysis summarized in (Figure 2b). Nucleolar IDRs show the highest number of significantly enriched IDRs with distinct compositions and blocky sequence patterns. Features that are enriched in nucleolar IDRs include high fractions of Lys residues, as well as large, linear blocks of charged residues. Recent work highlighted two main IDR grammars that set nucleolar proteins apart from the proteins of all other condensates (Figure 2c,d). The first of these grammars is referred to as D/E-tracts and is defined by the presence of long, uninterrupted tracts of Asp (D) and Glu residues (E) (Figure 2c). The second grammar, referred to as K blocks + E-rich-regions, is defined by the presence of blocks of Lys (K) residues interspersed by Glu residues (Figure 2d).

Known nucleolar scaffolds contain some of the highest scoring D/E-tracts not only in the nucleolus but also in the human IDRome. NCL contains the highest scoring D/E-tract in the human IDRome and has been implicated as a scaffold for DFC formation. Similarly, UBF, which is necessary for FC formation, contains the third highest scoring D/E-tract in the nucleolus and fifth highest scoring D/E-tract in the human IDRome. Additionally, NPM1, which is a key marker and scaffold of the GC, contains the fourth highest scoring D/E-tract in the nucleolus and 16^th^ highest scoring D/E-tract in the human IDRome. These results, uncovered using a systematic and unbiased approach [46], suggest that condensate driving scaffold proteins may be identified by extracting proteins containing IDRs with condensate-specific, high scoring grammars.

Proteins containing K blocks + E-rich-regions have also been shown to promote nucleolar condensation, particularly of the FC [46]. Furthermore, it is now well established that IDRs featuring blocks of basic residues, especially Lys residues, promote partitioning into the nucleolus [140]. Consistent with the observation that essential FC and DFC factors have exceptionally high scoring K-blocks + E-rich-regions, it has been shown experimentally, both *in vivo* and *in vitro*, that z-scores of K-blockiness correlate positively with partitioning into the FC and DFC [46,112].

POLR1F and POLR1G, two subunits that are specific to RNA polymerase I, contain the highest scoring K blocks + E-rich-regions in the nucleolus, and are the first and fifth highest scoring K-blocks + E-rich-regions in the entire human IDRome. The unique subunits of the three different eukaryotic RNA polymerases provide specificity in transcription, determining whether they transcribe rRNA genes (governed by RNA polymerase I), mRNA, miRNA, snRNA, and snoRNA genes (governed by RNA polymerase II), or tRNA and 5S rRNA genes (governed by RNA polymerase III) [141]. It appears that IDRs within polymerase-specific subunits of RNA polymerases I, II, and III may also contribute to specificity via a grammar that encodes localization signals for the correct nuclear condensate in which transcription occurs [142].

Both the observed enrichment of proteins with D/E-tracts and K blocks + E-rich-regions in the nucleolus and recent experimental evidence suggests that complex coacervation plays a role in nucleolar assembly. Complex coacervation is a form of phase transition that is driven, in part, by polyelectrolyte complexation. Macromolecules of opposite charge combine via charge complexation, and segregate from solution to form charge-balanced dense phases known as coacervates [11]. Nucleoli in *Xenopus* oocyte germinal vesicles are enlarged when high doses of mRNA encoding for proteins containing D/E-tract and K blocks + E-rich-regions are introduced and conversely they dissolve when salt concentration is artificially increased within germinal vesicles [27,46]. These observations of nucleolar expansion and dissolution are consistent with complexation and coacervation among charge complementary macromolecules contributing to the stabilization of nucleoli. Interestingly, the deletion of two K blocks + E-rich-regions is sufficient to destabilize nucleoli in yeasts to the extent that they are no longer distinct entities in TEM images [143].

A striking feature of the compositional biases of nucleolar IDRs is the paucity of Arg residues, especially in the FC. The nucleolar exclusion of Arg-rich IDRs in non-ribosomal proteins first came to light in the work of Greig et al., who showed that IDRs with alternating Arg and acidic residues (Asp or Glu) are features that determine preferential localization to nuclear speckles [112]. Conversely, when Arg is replaced with Lys in these IDRs and the patterning is made more blocky, nucleolar accumulation results. Recent biophysical studies have shed light on the differences between Lys and Arg, which highlight why these residues, despite being cationic, are not interoperable with one another, especially in the context of IDRs where the consequences of differences in physical chemistry are significantly amplified [138,144,145]. The hydration properties, the basicity, the intrinsic secondary structure preferences, the context-dependent ion binding, and the interactions with nucleic acid substrates mark Arg and Lys as being distinct from one another [146]. These differences appear to point to a selection for the arginine deficient internal microenvironments of nucleoli, especially the FC [46] and the Arg-rich microenvironment of nuclear speckles, especially the regions enriched in SR containing proteins [91].

Lastly, it is worth noting that many of the nucleolar proteins that contain either of the two main IDR molecular grammars also feature substrate-binding domains that are tethered to the IDRs. For example, many of the nucleolar proteins that feature high-scoring D/E-tracts also include DNA and/or RNA binding domains [46]. The types of substrate-binding domains in these proteins determine the intra-nucleolar and sub-phase localization. For example, UBF has six high mobility group (HMG) domains which interact with rDNA in the FC [147,148], whereas the DFC-resident protein NCL interacts with pre-rRNA through its four RNA Recognition Motifs and RG-rich IDR [102,149–151] (Figure 1f-g). These results highlight the need to consider IDRs within their protein contexts given that the interplay between IDRs and substrate-binding domains can control proper localization of condensation.

Overall, the results gleaned to date suggest that an unbiased and systematic analysis of molecular grammars of IDRs that are condensate-specific can help identify sequence features that contribute to condensate formation, stabilization, and localization. IDRs are conformationally heterogeneous entities, with distinctive sequence features. Accordingly, and in the same way that flexible synthetic polymers engender distinctive material properties and microenvironments that lead to their deployment in a variety of industrial applications, we propose that the distinctive IDRs of nucleoli and other condensates enable the creation of condensate-specific microenvironments that function as distinct microreactors.

## Compositional specificity and IDR grammars generate distinct microenvironments

We have highlighted the macromolecular make-up of the FC, DFC, and GC condensates and pointed out that condensate composition correlates with the specific biochemical reactions that occur within the sub-phases (Figures 1 and 2). Next, we ask how the nucleolar compositional biases are linked to function? Undoubtedly, this question can be answered using a classical structure-function understanding of the macromolecular complexes involved in each biochemical reaction. To complement these approaches, we focus on the fact that condensation combined with compositional specificity generates distinct microenvironments, and that these are likely to have specific functional consequences.

**Figure 3.**
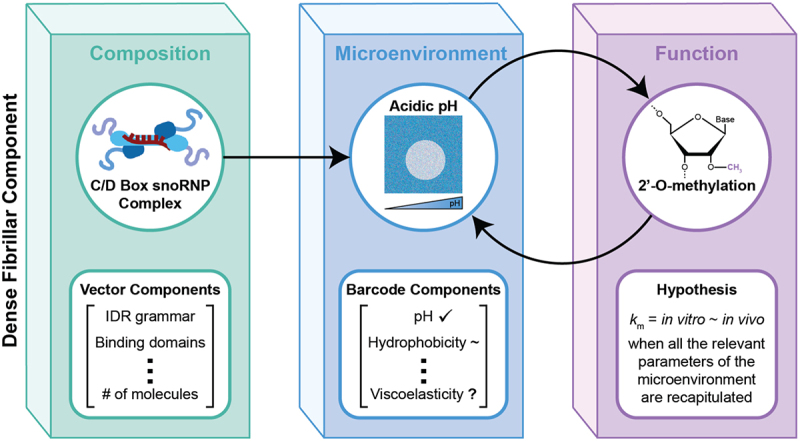
Example schematic of the relationship between composition, microenvironment, and function of the DFC. generally, the composition of a particular condensate can be described by a vector that includes IDR grammar features, binding domains, number of molecules, etc. The composition of the condensate then dictates the microenvironment, which is defined by a barcode of features including pH, hydrophobicity, and viscoelasticity, and the function of the condensate. Specifically, the compositional features of the C/D Box snoRNP Complex, including the RG-rich IDR, the K block + E-rich regions IDRs, and the RNA-binding domains, contribute to the unique microenvironment and function (2’-O-methylation) of the DFC.

The functional importance of microenvironments can be illustrated by homing in on a specific biochemical reaction, in this case 2'-O-methylation, which is essential for pre-rRNA maturation (Figure 3). Ribosomal RNA is significantly stabilized by being 2'-O-methylated in roughly 90 positions along its sequence [152]. This biochemical process is carried out by the C/D box snoRNP which comprises the S-adenosyl-L-methionine-dependent methyltransferase Fibrillarin (FBL), as well as a Nop dimer (comprising homodimers of Nop58 or Nop56, or a heterodimer of the two) and two Snu13 proteins. Studies on purified C/D box snoRNPs from thermophilic yeasts have helped establish that these complexes have structural versatility that could aid targeting. Nonetheless these complexes exhibit limited and inaccurate 2'-O-methylation activity *in vitro* [153–155]. One recent study showed that purified C/D box snoRNP facilitated incomplete 2'-O-methylation over the course of 30 minutes and was inaccurate in methylating its target nucleotide, whereas *in vivo* this process is thought to occur with precision on much shorter timescales [156]. Unlike the *in vivo* reality, the functional studies in reconstituted systems are carried out with snoRNPs lacking IDRs, and the components were assembled in dilute, mono-dispersed solutions at a pH of 7.0–7.4. Indeed, it is common for biochemical processes under such conditions to take orders of magnitude longer *in vitro* and improvements often entail optimizations that account for details of missing factors (e.g., IDRs and conditions that mimic internal microenvironments).

The compositions of nucleolar sub-phases, like the DFC, directly dictate their microenvironment (Figure 3). We propose that microenvironments are best described using physicochemical barcodes that quantify properties such as internal pH overall water content, interphase activities of ions and metabolites, electronic susceptibilities such as dielectric responses, internal viscosity, frequency-dependent viscoelastic moduli, and internal hydrophobicity. Interphase electrochemical potentials and susceptibilities, which enable the storage of charge, metabolites, and protons, will give condensates the characteristics of induction motors that enable force generation and sensing through viscocapillary effects [116,157]. The presence of proteins with D/E tracts, a distinctive feature of nucleolar proteins, sets them up to be carriers of protons [46]. This is because long D/E tracts are likely to extract entropic penalties associated with organizing water molecules around ionic moieties [158]. Further, the high linear charge density engenders intramolecular electrostatic repulsions that extract penalties in terms of conformational entropy [159,160]. These entropic penalties can be offset by neutralizing some of the charge, and the most efficient way to achieve this is through proton binding, which leads to upshifts of pK_a_ values of acidic groups – a sequence-encoded emergent property known as *charge regulation* [160,161]. An additional outcome of pH- and context-dependent charge regulation, which refers to selective protonation of acidic groups and deprotonation of basic groups, is that it generates a heterogeneity of charge states [159] which should enable a diverse set of interaction modes.

Recent studies have shown that features such as D/E tracts that are prominent in nucleolar proteins enable these moieties to function as proton carriers that set up a gradient whereby proton concentrations are highest within the FC/DFC and decrease within the GC, [46,162]. A proton gradient generates an acidic microenvironment for nucleoli, and it can be used as a proton motive force for enabling the outward flux of rRNA and inward flux of ribosomal proteins [47,163]. The compositional biases also set up strong enough differences in overall hydrophobicity/hydrophilicity of specific mixtures relative to one another, thus ensuring differential solvation profiles and hence differential interfacial free energies that enable the organization of nucleoli into distinct layers [27]. It is noteworthy that the differences in solvation properties are strong enough to enable the formation of coexisting phases, but weak enough to ensure that the layer-specific components do not end up forming *de novo* condensates. The specificity of co-condensation and differences in composition-specific solvation properties engender the typical FC-DFC-GC layering, although this is sensitive to the levels of different nucleic acid substrates *viz*., rDNA, pre-rRNA, and mature rRNA (mat-rRNA) [3].

## Evolutionary considerations

**Figure 4.**
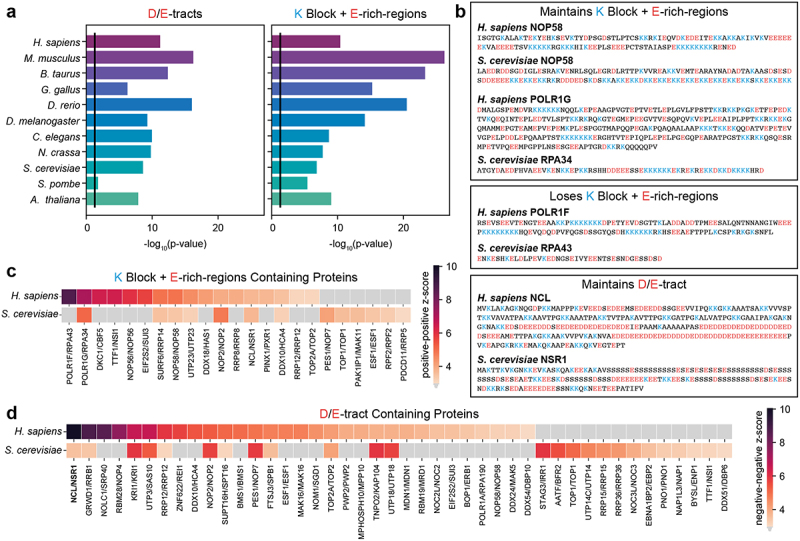
D/E-tract and K block + E-rich-regions IDRs are conserved. (a) p-values quantifying the enrichment of D/E-tract and K block + E-rich-region containing IDRs within nucleolar proteins of 11 well-studied eukaryotic species (Fisher’s exact test). The black line denotes a p-value cutoff of 0.05. Nucleolar proteins were extracted from the human protein atlas for *H. sapiens* and DrLLPS for all other species. (b) Example IDR sequences of human and yeast homologous pairs that either maintain or lose the given IDR feature across evolution. (c-d) the conservation of specific proteins maintaining K block + E-rich-regions (c) and D/E-tract (d) IDRs across evolution. Homologs were identified using YeastMine. Positive-positive (c) and negative-negative (d) z-scores are shown, where a gray box denotes a z-score <3 indicating that IDR feature is lost for that protein. Gene names are given with the human name followed by the yeast name.

Thus far we have focused on nucleolar features in higher eukaryotes. Do the molecular grammar features and microenvironment parameters persist across all eukaryotes? Analysis of nucleolar proteins in the DrLLPS database (https://llps.biocuckoo.cn/) shows that IDRs containing D/E-tracts and K-blocks + E-rich-regions are enriched across 11 well-studied eukaryotic species (Figure 4a). This suggests that the molecular grammars that characterize nucleolar scaffolds, as gleaned from analysis of higher eukaryotic species, are conserved across eukaryotes, including in plants and fungi. For example, NCL appears to be a key scaffold for the formation of the DFC in higher eukaryotes and its D/E-tracts helps drive condensation. Figure 4(b) shows that the presence of D/E-tracts in NCL is conserved in its yeast homolog, NSR1. In contrast, RPA43, the yeast homolog of POLR1F, the protein with the highest scoring K-blocks + E-rich-regions IDR in the human proteome, does not contain the characteristic grammar of K-blocks + E-rich-regions (Figure 4b). Thus, our analysis shows that even though the two dominant IDR grammars of the nucleolus are conserved across eukaryotes, the exact proteins that contain these grammars may change (Figure 4c,d). From proteins within the YeastMine homolog set [164] 31 human nucleolar proteins and 25 yeast nucleolar proteins were found to contain D/E-tract IDRs. However, only 12 pairs of protein homologs contain D/E-tracts in both yeasts and humans. Similarly, from proteins within the YeastMine homolog set, 17 human nucleolar proteins and 13 yeast nucleolar proteins contain K-blocks + E-rich-regions, but only 7 pairs of protein homologs contain these IDRs in both yeasts and humans. Of interest is NOP58, a key protein of C/D box snoRNP complexes. Colau et al., showed that the deletion of the K-block + E-rich-region of NOP58, as well as the somewhat less blocky K- and E-rich-region of NOP56, disrupted nucleolar morphology in yeast [165]. Specifically, upon deletion, the nucleoli became more homogeneous, and the nucleolar volumes increased. This suggests that IDR grammars identified in higher eukaryotic species that provides cohesive forces for hierarchical organization, are also important for nucleolar organization and compaction in yeast.

Interestingly, while not all the key proteins maintain the dominant grammar, the grammars are preserved across the essential complexes (Figure 1 f). In the FC, K-blocks + E-rich-regions and D/E-tracts are found in protein complexes that are directly involved in rDNA transcription, including Topoisomerase I, the transcriptional activation complexes, and RNA polymerase I. For example, while RNA polymerase I contains the highest scoring Kblock + E-rich-region in yeast, this IDR is found in RPA34 (POLR1G in humans) rather than RPA43 (POLR1F in humans) (Figure 4b,c). Similar observations can be made regarding IDRs of essential DFC complexes – C/D box and H/ANA box snoRNPs that contribute to 2' methylation and pseudo uridinylation, respectively.

## Conclusions

Considerable progress is being made toward compositional profiling of condensates. These efforts are yielding detailed inventories of macromolecules that are within condensates and the relative abundance of the underlying components. Proteomic atlases are also providing information regarding the spatial profiles of macromolecular components both within and outside condensates. These data clearly point to inhomogeneous organization of the underlying macromolecules and the inhomogeneities extend to length scales that are at least an order of magnitude larger than macromolecular dimensions. These types of compositional and mesoscale spatial/density inhomogeneities should give rise to emergent physicochemical properties within condensates. We propose that the distinctive compositions and inhomogeneous organization of constituent macromolecules of condensates give rise to condensate-specific interphase properties that can be summarized in terms of unique, condensate-specific *physicochemical barcodes*. These barcodes quantify the electrochemical, electrical, mechanical, hydrodynamic, and biochemical properties of condensates.

The nucleolus is the most prominent biomolecular condensate in cells. Detailed compositional profiling and spatial maps coupled to analysis of molecular grammars have yielded insights regarding the types of molecules that are enriched within different nucleolar regions. These biases, particularly the presence of D/E tracts within many nucleolar proteins, led to the prediction and subsequent demonstration that the D/E tracts are proton carriers that generate a proton gradient between the nucleolus and the surrounding nucleoplasm [46]. The links between biased composition and emergent physicochemical properties remain largely uninvestigated, with the only other property similarly examined being the relative hydrophobicities of FC versus DFC and the DFC versus the GC [27,46]. In this sense, the barcoding of nucleoli in terms of physicochemical barcodes has focused, to this point, only on a couple of bars in the barcode. What is required are measurements of other electrochemical, electrical, mechanical, hydrodynamic, and biochemical properties both *in vitro* and in live cells. This will require the use of either direct measurements or the deployment of multiplexed probes whereby optical, fluorescent, or other signals serve as proxy reporters of physicochemical barcodes. Such efforts will be essential, both for connecting physical principles to functions that operate on mesoscales within cells and for understanding how specificity on the molecular scale enables specificity of emergent properties. Condensation generates distinct collectives of macromolecules, and emergent properties are properties of collectives. Therefore, it stands to reason that distinct measurements will be needed to quantify how composition- and grammar-driven emergent properties give rise to distinct microenvironments for condensates such as nucleoli.

## Data Availability

Data sharing is not applicable to this article as no new data were created or analyzed in this study.
